# Expansion of Circulating Tumor Cells from Patients with Locally Advanced Pancreatic Cancer Enable Patient Derived Xenografts and Functional Studies for Personalized Medicine

**DOI:** 10.3390/cancers12041011

**Published:** 2020-04-20

**Authors:** Lianette Rivera-Báez, Ines Lohse, Eric Lin, Shreya Raghavan, Sarah Owen, Ramdane Harouaka, Kirk Herman, Geeta Mehta, Theodore S. Lawrence, Meredith A. Morgan, Kyle C. Cuneo, Sunitha Nagrath

**Affiliations:** 1 Department of Chemical Engineering, University of Michigan, Ann Arbor, MI 48105, USA; lianette@umich.edu (L.R.-B.); gogodidi@umich.edu (E.L.); snowen@umich.edu (S.O.); 2 Biointerfaces Institute, University of Michigan, Ann Arbor, MI 48105, USA; mehtagee@umich.edu; 3 Department of Radiation Oncology, University of Michigan Medical School, Ann Arbor, MI 48109, USA; ines.lohse@gmail.com (I.L.); kherman@med.umich.edu (K.H.); mmccrack@med.umich.edu (M.A.M.); 4 Department of Materials Science and Engineering, University of Michigan, Ann Arbor, MI 48109, USA; shreyar@umich.edu; 5 Department of Internal Medicine, University of Michigan Comprehensive Cancer Center, Ann Arbor, MI 48109, USA; rharouaka@gmail.com (R.H.); tsl@umich.edu (T.S.L.); 6 Veterans Administration Ann Arbor Healthcare System, Ann Arbor, MI 48105, USA

**Keywords:** circulating tumor cells, biomarkers, pancreatic cancer, personalized medicine

## Abstract

Improvement in pancreatic cancer treatment represents an urgent medical goal that has been hampered by the lack of predictive biomarkers. Circulating Tumor Cells (CTCs) may be able to overcome this issue by allowing the monitoring of therapeutic response and tumor aggressiveness through ex vivo expansion. The successful expansion of CTCs is challenging, due to their low numbers in blood and the high abundance of blood cells. Here, we explored the utility of pancreatic CTC cultures as a preclinical model for treatment response. CTCs were isolated from ten patients with locally advanced pancreatic cancer using the Labyrinth, a biomarker independent, size based, inertial microfluidic separation device. Three patient-derived CTC samples were successfully expanded in adherent and spheroid cultures. Molecular and functional characterization was performed on the expanded CTC lines. CTC lines exhibited KRAS mutations, consistent with pancreatic cancers. Additionally, we evaluated take rate and metastatic potential in vivo and examined the utility of CTC lines for cytotoxicity assays. Patient derived expanded CTCs successfully generated patient derived xenograft (PDX) models with a 100% take rate. Our results demonstrate that CTC cultures are possible and provide a valuable resource for translational pancreatic cancer research, while also providing meaningful insight into the development of distant metastasis, as well as treatment resistance.

## 1. Introduction

Pancreatic cancer is the third leading cause of cancer-related death in the United States [1]. The survival probability with this disease has not improved substantially over nearly 40 years [1,2]. While surgical removal of the tumor represents the best treatment option for pancreatic cancer patients, only 20% of patients qualify for surgery [2,3]. Chemotherapy or chemotherapy combined with radiation is typically offered to patients with locally advanced disease [3,4].

A major challenge in the management of these patients is the early assessment of response to therapy that would allow for the selection of the appropriate therapy and limit toxicity in treatment-resistant patients. Computed tomography (CT) is routinely used to stage and reassess patients following treatment. However, a number of studies have demonstrated that CT-detected treatment responses are infrequent [5]. Obtaining tissue from pancreatic cancer patients with locally advanced disease for histological diagnosis and acquiring pre- and post-monitoring presents a substantial challenge.

Over the past few years, several studies have examined circulating tumor cells (CTCs) in many epithelial cancers and suggested that CTCs can be used as clinical biomarkers of treatment response and prognosis [6,7,8,9,10,11,12]. CTCs are cancer cells that have shed into the vasculature or lymphatics from a primary tumor, and are carried around the body in the circulation. CTCs are believed to have the potential to develop into distant metastases, which are the major cause of cancer related mortality. However, the isolation of viable CTCs is an area of active research with limited success to date. Hence, the lack of such technologies hamper culture approaches.

A widely established—and the only Food and Drug Administration (FDA) approved—approach for CTC isolation is the CellSearch system, which uses magnetic beads functionalized with antibodies against the epithelial cellular adhesion molecule (EpCAM) [12,13,14]. Due to the limitations of antibodies used for CTC capture, this system fails to detect cancer cells with reduced EpCAM expression, and may thus only enrich a subpopulation of CTC [15,16,17,18]. Hence, there was a thrust for the development of new technologies with higher sensitivity, as well as with the ability to isolate broader subpopulations of CTCs. This is being addressed by the use of microfluidic technologies that allow for the unprecedented spatio-temporal control of cells [10]. Their application in cancer research is now well established [19], with a number of studies demonstrating successful isolation and characterization of CTCs from clinical samples [20]. Microfluidic CTC isolation technologies are mainly categorized by their exploitation of either CTCs’ distinctive (i) biological properties, or (ii) physical properties [19]. The former is based on the expression of cell surface markers, while the latter includes size, deformability, density, and electric charge [21]. The use of CTCs’ physical properties to develop microfluidic devices allows label-free isolation, which overcomes biased cell selection using the CTCs’ biological properties, such as protein expression and molecular markers. Furthermore, isolated cells using label-free technology are not modified, which permits greater flexibility for downstream characterization of CTCs. In summary, advances in label free microfluidic technologies allow the reliable detection and isolation of CTCs from clinically available blood draws. This will, in turn, allow functional characterization of CTCs to understand the utility of CTCs as predictive and prognostic markers, and may serve as a surrogate tumor biopsy.

Although previously reported clinical studies have mostly focused on CTC enumeration in guiding prognosis in metastatic cancer patients, current research is exploring the pharmacodynamic and predictive biomarker utility of CTCs. A number of studies have isolated and evaluated CTCs in patients with pancreatic adenocarcinoma [22]. Early studies identified CTCs in pancreatic cancer patients with metastatic disease, using several tumor cell markers, including CK20, CEA, and c-MET, and demonstrated that, compared to other types of malignancies, these patients have relatively low numbers of CTCs [23,24,25,26,27]. Recent reports have concentrated on CTCs in patients with locally advanced pancreatic cancer. Ren et al. examined CTCs in 31 patients with stage III and nine patients with stage IV pancreatic cancer. Eighty percent of these patients were found to have CTCs prior to chemotherapy, and this number decreased to 29% after treatment [28]. Bidard et al. analyzed blood samples of 79 patients with locally advanced pancreatic cancer treated on a clinical trial for CTC detection. This study identified CTCs in only 11% of patients; however, the presence of CTCs was associated with poor outcome [12].

Beyond CTC enumeration, an ex vivo expansion and functional characterization of patient-derived CTCs will help to elucidate the clinical application of CTCs in pancreatic cancer. Due to their low frequency in pancreatic cancer patients [22], expanding such cells becomes a critical requirement of any CTC study. To our knowledge, no long-term CTC cultures in pancreatic cancer have been reported. However, some success has been reported across other types of cancers using affinity-based approaches. In breast cancer, CTC cultures were reported by Yu et al. using the CTC-iChip [29]. Cultures were generated under hypoxic and non-adherent culture conditions, achieving success for 6/36 breast samples. In colon cancer, Cayrefourcq et al. were also able to expand CTCs from 2/71 colon cancer patients [30]. Our own research group reported CTC culture in lung cancer, using a microfluidic co-culture device, where 14 out of 19 samples were expanded [31]. While these technologies have shown the ability to expand CTCs, the inherently biased CTC selection of immunoaffinity based technologies limits the ex vivo functionality study of all distinct subpopulations of CTCs.

In order to further validate the utility of expanded CTCs, researchers have started developing CTC-derived xenografts (CDX) models. Such models serve as a method to examine expanded CTCs’ in vivo properties, including tumorigenicity and drug susceptibility. For example, Cayrefourcq et al. showed the generation of colon tumors from a CTC-derived cell line in immunodefficient mice [30]. In breast cancer, Yu et al. successfully established five CTC cell lines, where three were tumorigenic in mice [29]. In small-cell lung cancer, Hodgkinson et al. demonstrated that CTCs from patients with either chemosensitive or chemorefractory tumors are tumorigenic in immune-compromised mice. Furthermore, their findings mirrored the donor patient’s response to platinum and etoposide chemotherapy [32]. In order to successfully perform these in vivo CTCs studies, a high initial concentration of CTCs was required (>10^6^ cells) for all studies, making CTC expansion essential.

Recently, we developed and optimized a high-throughput, label-free microfluidic device, the “Labyrinth”, for the size-based isolation of CTCs [33]. The Labyrinth has demonstrated greater than 90% recovery when tested with various cell lines, including pancreatic cancer cell lines, while achieving an 89% white blood cell (WBC) removal [33]. In our approach, since no positive or negative selection of cells is needed, the Labyrinth enables the study of CTC heterogeneity, and allows for the identification of multiple CTC subpopulations through further downstream biological and functional studies. Our previous study showed the presence of CTCs that have undergone the epithelial-to-mesenchymal transition (EMT) across all pancreatic cancer patients [33]. The use of our label-free Labyrinth device enables the study of viable EMT-like CTCs, which are believed to be the most aggressive subtype of CTCs [34,35,36]. Building upon our previous expansion work on lung cancer, this study uses the Labyrinth to expand CTCs using a monoculture approach to maintain the simplicity of the Labyrinth by eliminating the need of CTC purification from other cell lines.

In the present study, we explore the utility of pancreatic CTC cultures as a preclinical model for treatment response. CTCs were isolated from the blood of pancreatic cancer patients with locally advanced disease, using the Labyrinth and expanded in vitro. CTC cultures were then characterized in both 2D (adherent) and 3D (spheroid) conditions. Such characterization was performed through the examination for epithelial and EMT features and compared to the original patient specimen. Furthermore, we evaluated tumorigenicity in vivo, by injecting cultured CTCs into a Nonobese diabetic/severe combined immunodeficiency (NOD/SCID) mice, thus creating a pancreatic CDX model. To our knowledge, these are the first ever pancreatic CTC cell lines developed from pancreatic patient samples. The isolation and expansion of CTCs can provide meaningful information to elucidate the process of pancreatic tumorigenesis and dissemination to preempt its fatal result.

## 2. Materials and Methods 

### 2.1. Cancer Cell Line Culture

Panc-1 and H1650 cells were purchased from ATCC (Manassas, VA, USA). bxPC-3 and MIA PaCa-2 were gifted by Dr. Diane Simeone lab (University of Michigan, Ann Arbor, MI, USA). All cells were maintained at 37 °C in 5% CO_2_. Cells were grown to 70%–80% confluence, before subculturing using 0.05% Trypsin-EDTA (Thermo Fisher Scientific, Waltham, MA, USA). Between subculturing, media was replenished every 48–72 h. Panc-1 cells were grown in Dulbecco’s Modified Eagle Medium (DMEM) (Thermo Fisher Scientific), supplemented with 10% fetal bovine serum (FBS) (Sigma-Aldrich, St. Louis, MO, USA) and 1% Antibiotic-antimycotic (Thermo Fisher Scientific). MIA PaCa-2 cells were grown in in Dulbecco’s Modified Eagle Medium (DMEM) (Thermo Fisher Scientific), supplemented with 10% FBS (Sigma-Aldrich), 2.5% horse serum and 1% Antibiotic-antimycotic (Thermo Fisher Scientific). bxPC-3 and H1650 cells were grown in RPMI-1640 (Thermo Fisher Scientific), supplemented with 10% FBS (Sigma-Aldrich) and 1% Antibiotic-antimycotic (Thermo Fisher Scientific). Cells were routinely tested for and reported negative for mycoplasma contamination.

### 2.2. Patient Sample Collection

Whole blood for CTC isolation was obtained from ten patients with locally advanced pancreatic cancer, as part of an Institutional Review Board approved protocol (HUM00085016). Each patient’s blood was collected prior to the start of therapy, and from three of the patients, a second sample was collected at the end of the first cycle of chemotherapy. Prior to study enrollment, patients were confirmed to not have distant metastases and to not be resectable at a multidisciplinary tumor board. Informed consent was obtained from all participating patients.

### 2.3. CTC Isolation

Whole blood samples from pancreatic cancer patients were treated with 6% dextran (molecular weight 250,000) to achieve density separation of red blood cells. The sample was incubated with dextran solution for one hour, after which point sedimentation of red blood cells was observed. The supernatant was then collected and diluted 3× with phosphate buffered saline (PBS). The Labyrinth device was primed with 1% Pluronic acid solution (diluted in PBS) at 100 μL/min for 10 min, followed by a 10 min incubation, to prevent cell clotting on channel walls. The diluted supernatant containing the cells was then flowed through the device at a flow rate of 2 mL/min. To increase sample purity, the effluent from the second outlet (CTC outlet) was collected, diluted 2×, and flowed through the Labyrinth for a second time. This repeated processing has shown to improve purity from 1.78 log WBC depletion (range 1.49–2.23) to a log WBC depletion of 4.3 in average (range 3.9–4.5). The enriched CTCs were then characterized by immunofluorescence and expansion.

### 2.4. CTC Expansion

The enriched fraction of CTCs collected from outlet 2 was treated with RBC lysis buffer at a 2:1 (buffer:sample) and incubated on ice for 3 min. Samples were then spun down, and the pellet was resuspended in culture medium. Approximately 85% of the second outlet product was utilized for CTC expansion, while the rest was used for Day 0 CTC counts. The culture medium consists of RPMI1640 (Thermo Fisher Scientific), supplemented with 10% FBS (Corning, Corning, NY, USA) and 1% antibiotics (Thermo Fisher Scientific). For adherent cultures, the cell suspension was plated into fibronectin (10 µg/mL, Advanced Biomatrix #5050-1MG, San Diego, CA, USA) coated 24 well plates.

### 2.5. Iummunofluorescence Staining

CTCs collected from the second outlet of the Labyrinth were cytospun onto slides using Cytospin 4 Cytocentrifuge (Thermo Fisher Scientific), according to the manufacture’s guidelines. Cytoslides were fixed using 4% paraformaldehyde (PFA) and stored at 4 °C until further staining.

For immunostaining, samples were permeabilized with Phosphate Buffered Saline with 0.05% Triton (PBS-T) solution for 15 min and blocked using a 10% donkey serum for 30 min at room temperature (RT). Rabbit anti-human Vimentin (D21H3) XP® (1:500 Cell Signaling Technology, Danvers, MA, USA), Mouse IgG1 anti-human anti-Pan-Keratin (1:500 Biolegend, San Diego, CA, USA, 628601), Mouse IgG2a anti-human anti-Cytokeratin 19 (1:500 Santa Cruz sc-6278), and Rat IgG2b anti-human CD45 (1:500 Santa Cruz sc-70699) in blocking buffer were applied to samples overnight in a refrigerator at 4 °C. The next day, samples were washed for 5 min with PBS-T (3×). Donkey anti-rat AF FITC 488 (1:500 Life Technologies, Carlsbad, CA, USA, A21208), Donkey anti-mouse APC 647 (1:500 Life Technologies A31571), Donkey anti-rabbit 568 TxRed (1:500 Life Technologies A10042) were diluted in blocking buffer and applied to samples, which were incubated in the dark for 45 min at RT. Samples were again washed 3 times for 5 min with PBS. DAPI (1 µg/mL, Thermo Fisher Scientific) diluted in PBS was applied for 10 min at room temperature to label nuclei. A drop of Prolong® Diamond Antifade Mountant (Thermo Fisher Scientific) was then added and coverslips were mounted onto the slides for imaging.

### 2.6. CTC Spheroid Culture

Pancreatic cancer CTC lines were cultured in 10% fetal bovine serum and 1.5× antibiotics supplemented RPMI till 60%–70% confluency. Cells were removed from culture surfaces by gentle trituration and collected in conical tubes. Pancreatic CTC spheroids were generated on 384 well hanging drop array plates, following extensively established protocols [37,38,39,40]. Briefly, cell suspensions were adjusted such that 20 µL contained 100 pancreatic CTCs. Spheroids were initiated with 100 cells/drop, and allowed to aggregate and form within the hanging drop array matrix. Progression of spheroid formation was observed using live phase contrast microscopy, over a period of 14 days, with daily monitoring. Medium was supplemented every other day in 1–2 µL increments, to maintain a drop volume of 20 µL, as described previously. Calcein-AM and Ethidium homodimer staining was utilized to monitor viability of cells within pancreatic CTC spheroids, following protocols described previously [41]. Cells were imaged using an inverted Olympus IX-81 spinning disk confocal microscope (Olympus Life Sciences, Waltham, MA, USA), where the presence of green indicated live cells, and red fluorescence indicated dead cells.

### 2.7. CTC Spheroid Staining

Slides with spheroid sections were deparaffinized and rehydrated by dipping three times in xylene, two times in 100% ethanol and once each in 95% and 70% ethanol. Antigen retrieval was performed by boiling slides in citrate buffer (pH = 6.0) for 10 min. Chamber slides with cultured cells were fixed with 4% Paraformaldehyde (Electron Microscopy Sciences, Hatfield, PA, USA) for 10 min and then washed with PBS. Samples were then permeabilized with ice-cold 1:1 Methanol:Acetone for 1 min and washed with PBS. A blocking buffer consisting of 5% goat serum (Sigma-Aldrich, St. Louis, MO, USA) diluted in PBS was applied at room temperature for 30 min to prevent non-specific adhesion. Monoclonal anti-vimentin (5 µg/mL, Thermo Fisher Scientific: MA1-10459) was diluted in a blocking buffer and applied to samples overnight at 4 °C. Samples were washed 3 times for 5 min with PBS. Goat anti-mouse IgM CF770 (4 µg/mL, Biotium, Fremont, CA, USA: 20385), anti-Pan-Keratin Alexa Fluor 555 (0.64 µg/mL Cell Signaling Technology, Danvers, MA, USA: 3478S), anti-EpCAM APC (0.24 µg/mL BD Biosciences Franklin Lakes, NJ, USA: 347200), anti-CD44 BV510 (1 µg/mL, BioLegend, San Diego, CA, USA: 103043), and anti-CD45 FITC (1 µg/mL, BioLegend: 304005) were diluted in a blocking buffer and applied to samples overnight in a refrigerator at 4 °C. Samples were again washed 3 times for 5 min with PBS. DAPI (1 µg/mL, Thermo Fisher Scientific) diluted in PBS was applied for 10 min at room temperature to label nuclei. A drop of Prolong® Diamond Antifade Mountant (Thermo Fisher Scientific) was then added and coverslips were mounted onto the slides for imaging.

### 2.8. Flow Cytometry

Cells were trypsinized, washed with ice-cold PBS, and fixed at a concentration of 2 × 10^6^ cells/mL in ice-cold 70% ethanol. For γH2AX analysis, samples were incubated with a mouse anti–γH2AX-specific antibody (clone JBW301; Millipore, Burlington, MA, USA) overnight at 4 °C, followed by incubation with a fluorescein isothiocyanate–conjugated secondary antibody (Sigma-Aldrich), as previously described [37]. For quantification of γH2AX positivity, a gate was arbitrarily set on the control, untreated sample to define a region of positive staining for γH2AX of approximately 5%. This gate was then overlaid on the treated samples. Samples were stained with propidium iodide to measure total DNA content and analyzed on a FACScan flow cytometer (Becton Dickinson, Franklin Lakes, NJ, USA) with FlowJo software (Tree Star, Ashland, OR, USA).

### 2.9. Immunohistochemistry

Xenograft tumors were excised, fixed and paraffin embedded. Paraffin tissue sections were cut, dried and dewaxed. Endogenous peroxidase was blocked in 3% hydrogen peroxide for 10 min. Microwave antigen retrieval was carried out under pressure at 120 °C for 10 min in a 10 mM Citrate buffer, pH 6.0 using a T/T Mega microwave oven. Endogenous biotin was blocked in Vector’s biotin blocking kit, and then slides were labeled with primary antibodies to cytokeratin (DAKO, Glostrup, Denmark, AE1/AE3, 1:200) and Smad4 (Abcam, Cambridge, UK (ab40759), 1:200) overnight. Biotinylated anti-mouse IgG incubations were carried out followed by streptavidin biotin detection system (Signet Pathology System, Deham, MA, USA) for 30 min each. Immunoreactivities were revealed by incubation in Nova Red substrate (Vector Lab, Burlingame, CA, USA) for 5 min and counterstained in Mayer’s haematoxylin.

### 2.10. Cell Proliferation Assay

Cultured cells were seeded at a concentration of 500 cells/well in 100µL of RPMI media into 96 well plates. Cells were incubated at 37 °C and 5% CO_2_ for 1–6 days. Cell Proliferation Reagent WST-1 (Roche, Basel, Switzerland) 10 µL/well was added and incubated for 1 h and Plate shaken for 1 min on a shaker. The absorbance was measured at an emission wavelength of 450 nm, using a Synergy Neo multi-purpose plate reader (Biotek, Winooski, VT, USA).

For spheroid cultures, CTCs were seeded on to custom injection-molded 384 well hanging drop array plates with 10 cells/drop using serum supplemented RPMI. Live/dead staining using calcein and ethidium homodimer was performed at day 14. Spheroids were then images using confocal microscopy.

### 2.11. KRAS G12/G13 Screening Using Digital PCR

RNA was extracted from CTC lines using miRNeasy Mini Kit (Qiagen, Hilden, Germany). RNA concentration was measured by Nanodrop spectrophotometer. cDNA was prepared using SuperScript IV VILO with ezDNase (Invitrogen, Carlsbad, CA, USA) with a loading of 2000 ng of RNA per cDNA synthesis reaction, following the manufacturer’s protocol.

KRAS G12/G13 mutation status was assessed using ddPCR™ KRAS G12/G13 screening kit (Bio-Rad laboratories, Hercules, CA, USA) on the RainDrop digital PCR system (RainDance Technologies, Lexington, MA, USA) using an optimized protocol. In brief, 25 µL PCR reactions were prepared using 12.5 µL Supermix (provided) with primers and probes at a concentration of 432 nM and 120 nM, respectively (0.48×). Five nanograms of cDNA were loaded into the PCR reaction. The PCR reaction was loaded onto the Source chip (RainDance Technologies). The Source machine (RainDance Technologies) generates a theoretical 4 million 5 pL droplets in an oil emulsion. The emulsified PCR reaction is then amplified on the thermocycler (Bio-Rad laboratories), following the screening kit’s provided thermocycling protocol. The PCR-amplified sample was transferred to the Sense machine (RainDance Technologies), and the end-point fluorescent intensity of each droplet is measured. Fluorescent intensity thresholding controls were determined using cell line controls.

All purified RNA, cDNA, and PCR related reagents were handled in a PCR workstation (AirClean Systems, Creedmoor, NC, USA), to prevent nuclease contamination.

### 2.12. Treatments

Cultured cells were seeded at a concentration of 1000 cells/well in 100 µL of RPMI media into 96 well plates. Cells were incubated for 24 h at 37 °C and 5% CO_2_. Media was exchanged for Gemcitabine (0, 0.05, 0.1, 0.5, 1, 5 uM) or 5-FU (0, 1, 5, 10, 50, 100 uM), diluted in RPMI media. Cells were incubated for 24 h, the media was replaced, and cells were further cultured for 48 h. Cell Proliferation Reagent WST-1 (Roche) was added 10 µL/well and incubated for 1 h. Plate was then shake for 1 min on a shaker. The absorbance was measured at an emission wavelength of 450 nm, using a Synergy Neo multi-purpose plate reader (Biotek).

For radiation treatments, cells were treated with a single dose of 4 Gy at 60–80% confluency and fixed for flow cytometry 16 h after treatment. For gemcitabine treatments, cells were treated with 100 nM gemcitabine for 2 h, and fixed for flow cytometry 22 h after treatment.

### 2.13. Xenografts

The protocols for this study have undergone review and approval by the University of Michigan Committee on the Use and Care of Animals (UCUCA). All mice were obtained through the University of Michigan Unit for Lab Animal Medicine (ULAM). Animal experiments were carried out using protocols approved by the University Animal Care Committee (PRO00006457) under the guidelines of the Association for Assessment and Accreditation of Laboratory Animal Care.

For CTC xenografts, 10^6^ cells were injected subcutaneously into the flank of 4–5-week-old NOD/SCID mice. Tumors were grown until the humane endpoint of ~1 cm in diameter or distress due to ascites or metastasis. Tumor size was evaluated twice a week using calipers.

## 3. Results

### 3.1. Patient Cohort

All patients included in the protocol were treated at the University of Michigan Medical Center between July 2014 and October 2016. Each patient had locally advanced pancreatic cancer, as determined at a multi-disciplinary tumor board. All patients on the study signed informed consent and were prospectively enrolled on an Institutional Review Board (University of Michigan) approved protocol. Patients received induction chemotherapy with single agent gemcitabine followed by 5 weeks of radiation therapy (52.5 Gy in 25 daily fractions), with concurrent gemcitabine and additional adjuvant chemotherapy afterwards.

All patients presented with surgically unresectable, non-metastatic pancreatic cancer (Table S1) at the beginning of the study.

The patients displayed an age range common for pancreatic cancer patients (53–74 years of age), and were evenly distributed between males and females (Table S1). Blood samples were taken just prior to the start of the treatment. From three of the patients (patients 1, 2, and 4), a second blood sample was taken at the end of the first cycle of gemcitabine-based chemotherapy (3 weeks after starting therapy).

### 3.2. Isolation of Patient-Derived Pancreatic Circulating Tumor Cells

**Figure 1:**
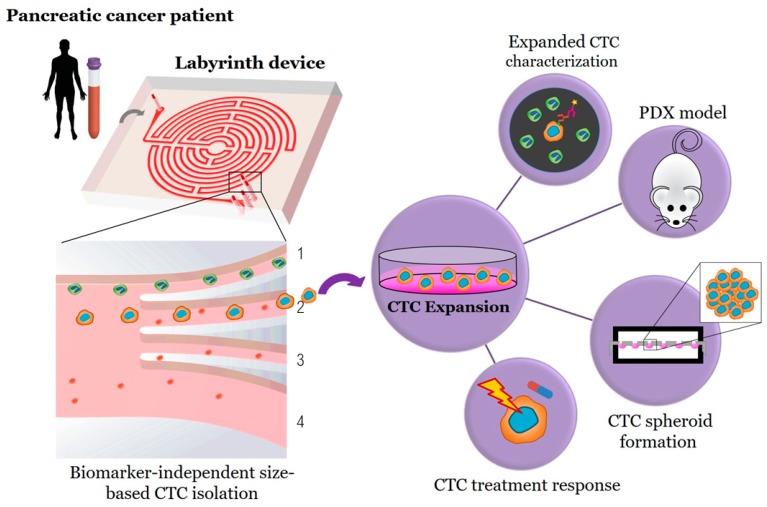
Workflow for biomarker independent isolation, expansion, and analysis of circulating tumor cells (CTCs). Through inertial focusing during Labyrinth processing, the CTCs (red cells) and WBCs (green cells) focus to unique streamlines. The WBCs focus to Outlet 1 and are depleted from the sample, while the CTCs are collected through Outlet 2 for further testing.

**Figure 2:**
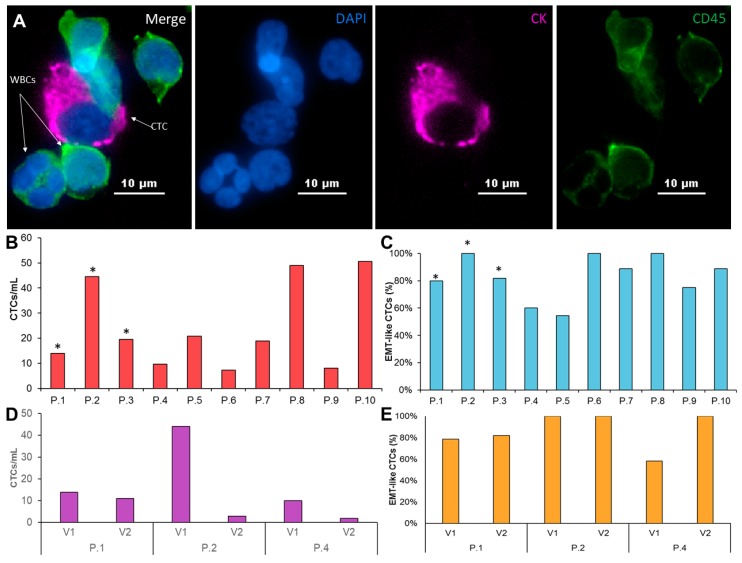
Characterization of patient-derived pancreatic CTCs. (**A**) A patient derived CTC (shown in pink), along with WBCs (shown in green) stained with CK 19, CD45, and DAPI. (**B**) CTC/mL enumeration for 10 PDAC patients. Asterisk marks the samples that were successfully expanded. (**C**) Coexpression percentage of cytokeratin and vimentin of CTCs isolated from 10 PDAC patients. (**D**) CTC/mL enumeration from two consecutive visits of 3 patients prior to treatment and after first round of chemotherapy. (E) two consecutive visits of 3 patients prior to treatment and after the first course of chemotherapy.

CTCs were isolated from whole blood based on size using the continuous high throughput label free Labyrinth device. The Labyrinth is an inertial microfluidic device with four outlets. As the sample is processed through the Labyrinth, cells of different sizes focus into separate streamlines across the channel, such that WBCs and CTCs each focus into distinct streamlines, which are collected through outlets 1 and 2, respectively (Figure 1) [33]. The isolated CTCs were divided in order to: (1) enumerate CTCs (~15% of isolated CTCs) and (2) set up a CTC culture (~85% of isolated CTCs). CTCs were identified in patient samples using the criteria of CK19+, CD45-, and DAPI+ (Figure 2A).

Similar to previous studies [22], we observed a wide range of CTC numbers from different patients. CTC numbers ranged from 8 CTCs/ml in patient 4 to 51 CTCs/ml in patient 10 (Figure 2B, Table S1). Since the EMT is believed to be essential for the generation of metastatic cells, we examined the presence of EMT-like and epithelial cells in enriched CTC samples (Figure 2C, Table S1). The majority of patients (7/10) show both EMT- like and epithelial cells in the purified CTC samples. Nevertheless, EMT-like cells represent the majority of CTCs. Only EMT-like cells were observed in the remaining 3 patients.

We obtained a second blood sample from three of the patients (patients 1, 2, and 4) after 1 cycle of chemotherapy. While the total CTCs/ml dropped for all three patients, we also observed patient-specific changes in the CTCs across these patients. The total number of CTCs/ml after chemotherapy dropped between 20–90%. Interestingly, patient 1, who retained the most similar CTCs/mL after one cycle of chemotherapy, also kept nearly the same epithelial and EMT-like fractions. Patient 2 showed a significant drop in CTCs/ml from 44 CTCs/ml to just 3 CTCs/ml and retained an entirely EMT-like phenotype before and after treatment. Patient 4 had an 80% reduction in CTCs/mL after one cycle of chemotherapy, but also showed a shift towards exclusively EMT-like CTCs after treatment. These patient-specific difference may suggest that CTCs can be used to study changes in response to treatment (Figure 2D,E, Table S2).

### 3.3. Circulating Tumor Cell Expansion and Characterization

Enriched CTCs of all 10 patients were seeded onto fibronectin coated 24 well plates to generate CTC-derived cell lines. We generated CTC-derived cell line from three individual patients (patients 1, 2, and 3). The majority of samples did not grow into stable CTC-derived cell lines. We observed an outgrowth of WBCs in one of the patients, while the remaining samples showed no growth or lost proliferation potential after the first passage. We did not find any correlation between stage (Table S1), CTC number (Figure 2B), or prior treatment with CTC growth in culture.

**Figure 3:**
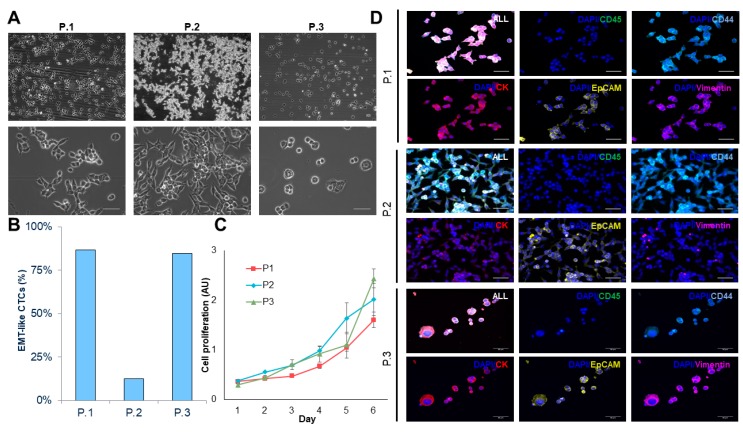
Characterizing CTC-derived cell lines. (**A**) Brightfield images (10× and 40×) of expanded CTC cultures. (**B**) Percentage of epithelial-to-mesenchymal transition (EMT)-like CTC in adherent cultures. (**C**) Growth curve analysis of adhered CTC cultures (n = 3). (**D**) Representative images of immunofluorescence staining using cytokeratin (red)/vimentin (magenta)/CD44 (light blue)/CD45 (green)/DAPI (blue). (Scale bar = 50 µm).

The three CTC-derived cell lines display distinctly different morphologies and EMT-like characteristics in culture that are consistent with the morphology observed in pancreatic cancer cell lines (Figure 3A,B). We observed differences in growth rate that remained stable over multiple passages (Figure 3C). Interestingly, the morphology of each CTC line didn’t correlate with EMT-related protein expression. Patients 1 and 3 had more cobblestone morphology, but had high vimentin expression, while patient 2 had the most spindle-like morphology, but had the lowest vimentin expression (Figure 3A,D, Table S3).

**Figure 4:**
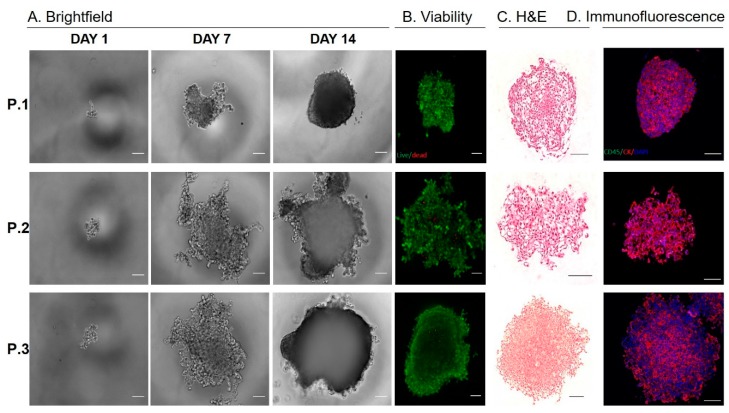
Characterizing CTC-derived spheroid cultures. (**A**) Pancreatic CTCs were seeded on hanging drop array plates with 10 cells/drop. Alamarblue fluorescence was used to monitor viability/proliferation (expressed as a fold increase at Day 7 and Day 14, compared to Day 1). (**B**) Representative images of live/dead staining using calcein and ethidium homodimer at Day 14, indicate high viability within pancreatic CTC spheroids. (**C**) Representative images H&E of spheroids. (**D**) Immunoflourescence staining for cytokeratin (red), CD45 (green) and DAPI (blue) on spheroids. (Scale bar = 100 µm).

Established CTC-derived cell lines were seeded on hanging drop array plates with 10 cells/drop in order to evaluate their ability to form viable spheroids (Figure 4A,B). All cell lines formed spheroids within 14 days, although we observed difference in growth rates across the three sample, which was similar to that of observed in the adherent cultures (Figure 3C). H&E sections of the spheroids showed a patient-specific morphology that is consistent with the range of morphologies observed in pancreatic cancer (Figure 4C). Interestingly, the spheroid cultures exhibited a shift in epithelial and EMT-like proportions, such that each population was more evenly represented, compared to adherent culture, but the dominate phenotype remained consistent (Figure 3B, Figure 4C,D, Table S3). This phenomenon was displayed in all three CTC lines, demonstrating the plasticity of these cells. The ability of these cells to alter their phenotype based on culture condition, may be informative in understanding how these cells adapt to different microenvironments throughout the metastatic cascade.

KRAS is mutated in the majority of pancreatic cancer patients, and the mutation contributes to the aggressive phenotype of the disease. KRAS mutations cluster in codons 12 and 13, and it has been suggested that the position and type of amino acid exchange influence the transforming capacity of mutant KRAS proteins. To screen the expanded CTCs for the presence of the KRAS G12/G13 mutations, we used digital PCR, which partitions the PCR reaction into millions of individual PCR reactions, with single molecule resolution. This assay simultaneously screens for seven KRAS G12/G13 mutations in parallel with wildtype KRAS. The pooled KRAS probes are labeled with the fluorophore FAM, while the wildtype probe is labeled with HEX. The expanded pancreatic CTCs were screened and compared to cell line controls. All three expanded CTC lines tested positive for a KRAS G12/G13 mutation (Figure S1). It was further observed that each of the CTC samples contained a heterozygous KRAS mutation, as shown by the presence of both wildtype and mutant populations (Figure S1).

### 3.4. Patient Derived Xengoraft Models from CTCs

We used a CTC patient derived xengroaft model (PDX) to investigate whether the CTC cell lines maintain the ability to form tumor in vivo that maintain the morphological characteristics of pancreatic tumors. The CTC derived cell line from patient 3 was injected into the flanks of 4–5 week old NOD/SCID mice.

**Figure 5:**
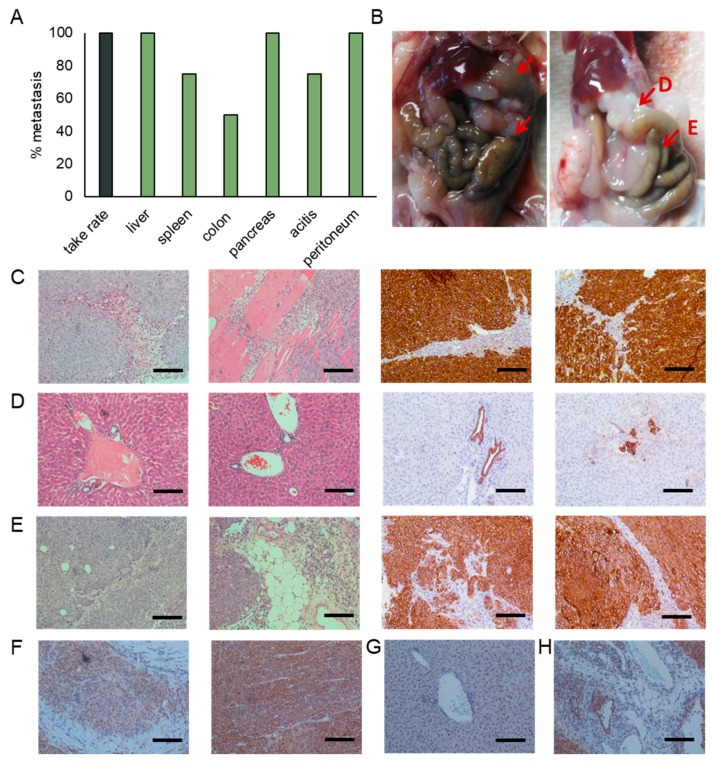
Characterization of the CTC patient derived xenograft (PDX). (**A**) Injection of the CTC cell line derived from patient 3 into the flanks of nonobese diabetic/severe combined immunodeficiency (NOD/SCID) mice resulted in the development of subcutaneous tumors in all injected mice. Additionally, we observed widespread metastases and the development of ascites. Percentages represent the percent of animals having metastasis to that site. (**B**) Representative images of mice showing metastases in the liver, peritoneum, colon and pancreas. Representative images of sections stained for cytokeratin and H&E of (**C**) subcutaneous tumors, (**D**) liver metastases, and (**E**) pancreatic metastases. Representative images of sections stained for Smad4 of (**F**) subcutaneous tumors, (**G**) liver metastases, and (**H**) pancreatic metastases. (Scale bar = 100 µm).

We observed the appearance of tumors in all injected mice 3–4 weeks after the injection of 10^6^ cells (Figure 5A). Tumors were harvested when the tumor size reached the humane end point. At that time, we observed widespread metastasis from the subcutaneous injection side to a number of organs, including the liver, peritoneum, and pancreas, as well as the development of ascites (Figure 5A,B). Immunohistochemistry shows cytokeratin positive subcutaneous tumors of pancreatic morphology that display large areas of tumor-associated stroma and invasion into the peritoneum (Figure 5C).

We observed the formation of macro (Figure 5B) and micro (Figure 5D) metastasis in the liver. While larger tumor masses were mostly observed on the surface of liver, a high number of micro metastases were observed throughout the entire liver, and were located near blood vessels.

Pancreatic tumor masses were observed in 100% of the injected animals. Although some pancreatic tissue remains, the majority of the pancreas has been overtaken by tumors (Figure 5E). These tumors display large areas of tumor-associated stroma that are characteristic for the desmoplasic reaction typically observed in pancreatic cancer.

Smad4 staining is observed throughout the subcutaneous tumors (Figure 5F). We do, however, observe a reduction in staining intensity in areas of pleural invasion. While Smad4 staining is lost in macro and micro metastases of the liver (Figure 5G), the expression is preserved in the pancreatic masses (Figure 5H). Similar to the subcutaneous tumors, staining intensity is reduced in areas of tissue invasion when compared to the tumor bulk.

### 3.5. CTC Cultures as a Surrogate for Treatment Response

The failure to predict treatment responses based on serum markers or biopsies is a major obstacle in the treatment of patients with pancreatic cancer. While the CTC numbers extracted from clinically available blood samples are too low to allow a direct analysis (Figure 2B), a high number of cells for the analysis of treatment responses can be obtained from low passage cultures.

**Figure 6:**
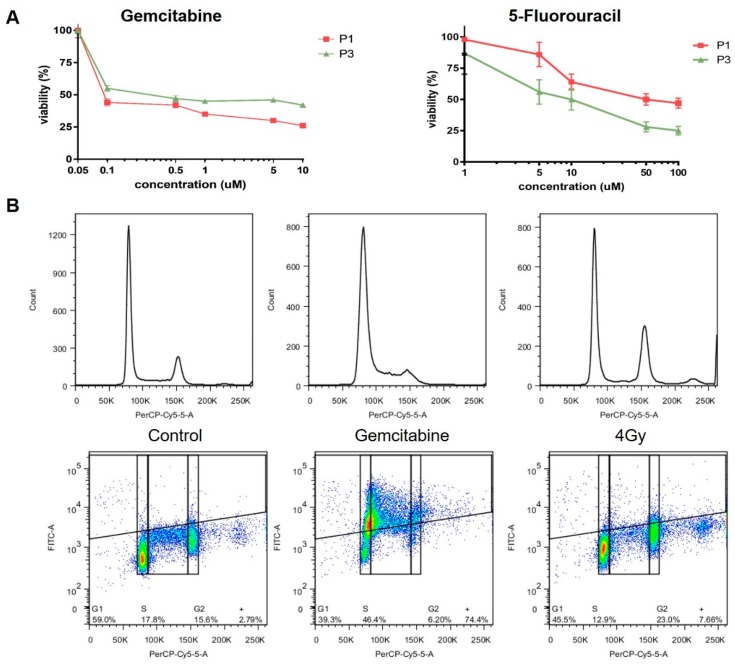
Treatment response of CTC cultures to chemotherapy. Response to treatment with (**A**) gemcitabine, 5FU and (**B**) IR was evaluated using Water Soluble Tetrazolium (WST) assays and flow cytometry, respectively.

The CTC cultures can be used for Water Soluble Tetrazolium (WST) assays, assessing proliferation in response to treatment, as demonstrated by treatment with gemcitabine and 5-Fluorouracil (5FU) (Figure 6A), as well as Flow cytometry based assays evaluating changes in cell cycle progression and accumulation of DNA damage in response to treatment with ionizing radiation (IR) or gemcitabine (Figure 6B). CTC cultures derived from patient 1 and 3 show significant differences in 5FU and gemcitabine sensitivity, which are consistent with a previously untreated pancreatic cancer cell. Treatment with gemcitabine resulted in the expected accumulation of CTCs in s-phase, while treatment with 4 Gy led to arrest in G2.

## 4. Discussion

Despite decades of extensive preclinical and clinical research, survival rates of patients with advanced pancreatic cancer have not improved significantly. The development of novel treatment approaches has been mostly hampered by the lack of available biomarkers, which can be used to assess the aggressiveness and metastatic potential and predictive/pharmacological markers of treatment response. Obtaining serial tissue biopsies to evaluate treatment response is not feasible in pancreatic cancer patients due to the invasive nature and risks associated with a biopsy. This may be overcome with CTCs, which can be safely obtained at any time point over the course of the treatment as part of routine blood draws.

Systematic evaluation of CTCs prior and during treatment will broaden our understanding of the biology of tumor aggression and metastasis, and ultimately improve treatment outcomes for pancreatic cancer patients. Beyond CTC enumeration, ex vivo expansion enable functional studies and drug screening for evaluating patient specific therapeutic targets. These patient-derived CTCs will also help to elucidate the presence and functional differences of small CTC subpopulations within the CTC pool. This can address one the critical challenges in treating pancreatic cancer; that is the ineffectiveness of drugs to treat the aggressive pancreatic cancer to eradicate cancer totally. Expanded CTCs will enable designing tailor-made treatments targeting all of the sub clones, instead of basing decisions solely on the predominant clone. Due to low CTC frequency in pancreatic cancer patients, especially in early stages, expanding such cells becomes a dire need of any CTC functional study [35]. While CTC cultures have been reported in the literature, including breast [29], colon [30], and lung [31], success rates are low. So far, no CTC cell lines have been reported in pancreatic cancer. We have previously reported culture of lung cancer-derived CTCs using a microfluidic co-culture device, where 14 out of 19 samples were expanded [31]. However, this approach limits the use of the cultured CTC for downstream applications, due to non-CTC cell contaminations. To overcome these limitations, the study presented here used a monoculture approach of patient-derived pancreatic CTC, isolated through the label free approach Labyrinth. Using the Labyrinth technology, we were able to isolate CTCs from 10 patients with locally advanced pancreatic cancer. The majority of the recovered cells were used to establish CTC cultures in vitro, while a small amount was used to evaluate and characterize the presence of epithelial- and EMT-like CTC sub populations.

We were able to isolate and evaluate CTCs from all patients enrolled in this study; however, we were only able to establish CTC cell lines from three of the patients. We observed the presence of both epithelial- and EMT-like populations, and found that EMT-like cells were significantly more abundant, pointing to the aggressive nature of pancreatic cancer. We found that each cell line retained a dominate phenotype regardless of 2D or 3D culture condition, however, some plasticity was observed. We are, however, cognizant of the fact that extended in vitro culture will likely result in the enrichment of single clones. Hence, for our experimental studies using CTC cultures, we restricted the usage to CTCs expanded within a maximum of 10 passages, which allowed us to perform several functional studies. However, it is important to note that we are able to grow these cells with repeated thaws and freezes beyond 10 passages. In our expanded CTCs, we observed distinct morphological differences associated with different phenotypes, ranging from epithelial to mesenchymal types, as well as significant differences in growth rate. Interestingly, we observed that CTC cell morphology was not predictive of molecular phenotype. For example, patient 2 exhibits mesenchymal-like cells with spindle like morphology (2D) and loosely packed spheroids (3D). However, the vimentin expression for this culture was substantially lower compared to the other two CTC cultures. This highlights the importance of several functional studies to achieve a better understanding of CTCs, rather than just few biomarkers by themselves.

In an effort to verify the pancreatic cancer identity of the three CTC-derived cell lines, we evaluated the KRAS mutation status of our cell lines and detected a heterozygous G12/G13 mutation in all three CTC cell lines.

Previous cancer studies have investigated the tumor initiating ability of CTCs in vivo [29,30,32]. However, none of these studies were performed on pancreatic cancer. This study demonstrates the first ever expansion of pancreatic CTCs into cell lines and the tumor initiating ability of the expanded CTCs in a mouse model. We attribute our success to the large number of CTCs we were able to isolate successfully using our label-free approach, which yielded CTCs with EMT characteristics. Consistent with the clinical phenotype of pancreatic cancer patients, the CTC-derived cell lines display rapid tumor growth in vivo. We observed widespread metastasis in mice injected with CTC-derived cell lines that match the metastatic sites that are commonly found in patients, such as liver and bowel, as well as the formation of ascites. These results further support the importance of CTC models in enhancing our understanding of the metastatic process in pancreatic cancer.

Treatment options are very limited for pancreatic cancer patients, and they typically receive treatment based on their tumor stage, rather than using precision medicine approaches due to the lack of predictive markers of treatment response. We believe that CTC cultures may be able to fill this gap by allowing for preclinical testing of newly developed compounds or personalized treatment approaches in a clinical setting. We have evaluated the utility of two of the most commonly used methods for in vitro drug sensitivity testing. Our CTC lines performed well in MTT cell toxicity assays and flow cytometry analysis of DNA damage and cell cycle progression, suggesting that these cultures can be used in range of cytotoxicity assays. These assays may be adapted to high throughput assays and used in personalized medicine approaches. Future studies will be necessary to evaluate the robustness of this system in a larger number of patient-derived CTC lines, and to evaluate the genetic and phenotypical stability of the cultures.

We had the opportunity to collect two consecutive samples from three patients. These samples were collected prior to the start of the therapy and after the first course of chemotherapy. While we were not able to establish CTC cell lines from consecutive visits, these samples enabled us to evaluate changes in the CTC phenotype in response to chemotherapy. Although both samples contained epithelial- and EMT-like populations of CTCs, we observed a trend towards increased numbers of EMT-like cells in response to treatment with gemcitabine. The small patient population enrolled in this study was not sufficient to further address this observation, or to make conclusions on the general population. Future studies in a larger cohort will aim to collect multiple samples throughout the patients’ treatment cycle, and are currently in the planning/enrollment stage. These studies will allow us to follow changes in the CTC phenotype in response to different treatment regimen, and correlate it to patient response and outcome. Once completed, these studies will shed further light on the importance of pancreatic CTCs in tumor metastasis, and the impact of anti-cancer therapy on CTC populations.

## 5. Conclusions

In this study, we demonstrated the ability to isolate and expand CTCs from pancreatic cancer patients isolated using the Labyrinth. CTCs were isolated from 10 locally advanced, treatment naïve pancreatic cancer patients, and we observed a wide range of CTCs/ml across these patients, as well as differences in the proportion of EMT-like and epithelial-like CTCs. From three of the patients, samples were collected pretreatment and after one cycle of chemotherapy. Patients tended to have fewer total CTCs/ml, as well as a higher proportion of EMT-like CTCs following chemotherapy, suggesting an enrichment for an aggressive phenotype.

CTC cell lines were established from three patients using a simple, 2D monoculture approach. These CTC-derived cell lines are compatible with traditional molecular, in vitro, and in vivo assays. We were able to identify patient-specific disease characteristics such as phenotype, mutation status, and treatment susceptibility. The CTC cell lines retained similar proportions of EMT-like and epithelial-like CTCs compared to the original sample, while also providing enough cell numbers for other characterization, not otherwise possible. The CTC cell lines showed differences in drug sensitivity when tested with gemcitabine and 5FU, highlighting the potential to use this platform for drug screening to identify which treatment a patient will likely respond to.

While the work presented in this study presents a small cohort of 10 patients, initial results suggest the ability to expand CTCs for personalized drug testing, and to monitor responses to treatment that may not be detected by current methods.

## Supplementary Materials

The following are available online at https://www.mdpi.com/2072-6694/12/4/1011/s1, Figure S1: KRAS G12/G13 mutation detection in CTC-derived cell lines, Table S1: Pancreatic cancer patient characteristics, Table S2: CTC number and characteristics change in response to treatment, Table S3: CTC characteristics in CTC cultures.
