# Supplementary material for: Expansion of Circulating Tumor Cells from Patients with Locally Advanced Pancreatic Cancer Enable Patient Derived Xenografts and Functional Studies for Personalized Medicine
Source: Cancers (Basel). 2020 Apr 20;12(4):1011. doi: 10.3390/cancers12041011 (PMC7225920; doi:10.3390/cancers12041011)
Supplement: Supplementary file 1 [file cancers-12-01011-s001.pdf]

# Supplementary Materials:

# Expansion of Circulating Tumor Cells from Patients with Locally Advanced Pancreatic Cancer Enable Patient Derived Xenografts and Functional Studies for Personalized Medicine

**Lianette Rivera-Báez, Ines Lohse, Eric Lin, Shreya Raghavan, Sarah Owen, Ramdane Harouaka, Kirk Herman, Geeta Mehta, Theodore S. Lawrence, Meredith A. Morgan, Kyle C. Cuneo and Sunitha Nagrath**

**Table 1.** Pancreatic cancer patient characteristics.

| Patient | Sex | Age | Diagnosis | Stage | CTC Number/mL | EMT-Like CTC/mL | Epithelial-Like CTC/mL |
|---|---|---|---|---|---|---|---|
| P.1* | m[#] | 53 | PDAC[$] | T4N0M0[**] | 14 | 11 | 3 |
| P.2* | f[##] | 74 | PDAC | T4N0M0 | 44 | 44 | 0 |
| P.3* | f | 54 | PDAC | T4N0M0 | 20 | 16 | 4 |
| P.4 | m | 64 | PDAC | T4N0M0 | 10 | 6 | 4 |
| P.5 | m | 53 | PDAC | T4N0M0 | 21 | 11 | 9 |
| P.6 | f | 73 | PDAC | T4N0M0 | 7 | 7 | 0 |
| P.7 | f | 60 | PDAC | T3N0M0 | 19 | 17 | 2 |
| P.8 | f | 69 | PDAC | T4N0M0 | 49 | 49 | 0 |
| P.9 | m | 61 | PDAC | T4N0M0 | 8 | 6 | 2 |
| P.10 | f | 50 | PDAC | T4N0M0 | 51 | 45 | 6 |

* CTC cell lines were established for these patients, # male, ## female, $ pancreatic ductal adenocarcinoma, **TNM classification of tumors of the exocrine pancreas: T4, tumor extends directly into any of the following: stomach, spleen, colon, adjacent large vessels; N0, no regional lymph node metastasis; M0, no distant metastasis.

**Table 2.** CTC number and characteristics change in response to treatment.

| Patient | Visit | Treatment | CTC Number/mL | EMT-Like CTC/mL | Epithelial-Like CTC/mL |
|---|---|---|---|---|---|
| **P.1** | V1 | untreated | 14 | 11 | 3 |
| | V2 | Gemcitabine, AZD1778 | 11 | 9 | 2 |
| **P.2** | V1 | untreated | 44 | 44 | 0 |
| | V2 | Gemcitabine, AZD1778 | 3 | 3 | 0 |
| **P.4** | V1 | untreated | 10 | 6 | 4 |
| | V2 | Gemcitabine, AZD1778 | 2 | 2 | 0 |

**Table 3.** CTC characteristics in CTC cultures.

| | Adherent Culture | | Spheroid Culture | |
|---|---|---|---|---|
| **Patient** | **EMT-Like CTC (%)** | **Epithelial-Like CTC (%)** | **EMT-Like CTC (%)** | **Epithelial-Like CTC (%)** |
| **P.1** | 87% | 13% | 57% | 43% |
| **P.2** | 12% | 88% | 37% | 63% |
| **P.3** | 85% | 15% | 66% | 34% |

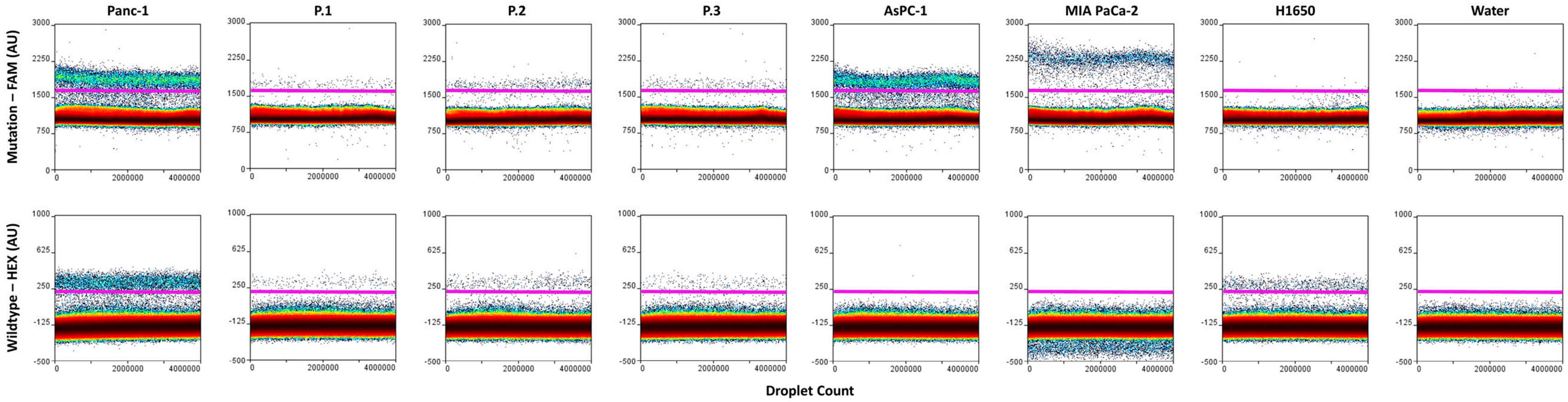


| | Panc-1 | P.1 | P.2 | P.3 | AsPC-1 | MIA PaCa-2 | H1650 | Water |
|---|---|---|---|---|---|---|---|---|
| Mutant KRAS | + | + | + | + | + | + | - | - |
| Wildtype KRAS | + | + | + | + | - | - | + | - |
| | Heterozygous | Heterozygous | Heterozygous | Heterozygous | Homozygous | Homozygous | Homozygous | N/A |

**Figure 1.** KRAS G12/G13 mutation detection in CTC-derived cell lines. Digital PCR plots of CTC-derived cell lines compared to control cell lines, with mutant (FAM) droplets (top) and wildtype (HEX) droplets (bottom) compared to droplet count. The corresponding KRAS G12/G13 mutation status is shown below.
